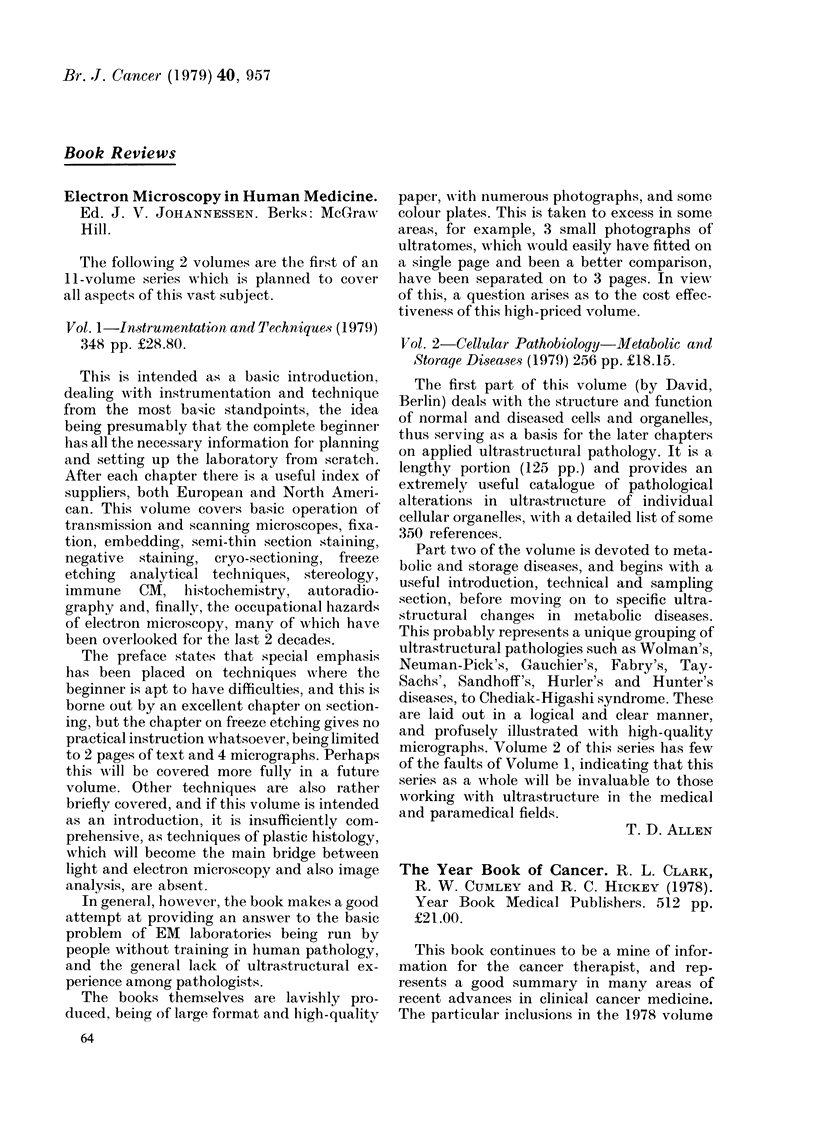# Electron Microscopy in Human Medicine

**Published:** 1979-12

**Authors:** T. D. Allen


*Br. J. Cancer* (1979) **40**, 957

### *Book Reviews*

**Electron Microscopy in Human Medicine.** Ed. J. V. JOHANNESSEN. Berks: McGraw Hill.

The following 2 volumes are the first of an 11-volume series which is planned to cover all aspects of this vast subject.

*Vol.* 1—*Instrumentation and Techniques* (1979) 348 pp. £28.80.

This is intended as a basic introduction, dealing with instrumentation and technique from the most basic standpoints, the idea being presumably that the complete beginner has all the necessary information for planning and setting up the laboratory from scratch. After each chapter there is a useful index of suppliers, both European and North American. This volume covers basic operation of transmission and scanning microscopes, fixation, embedding, semi-thin section staining, negative staining, cryo-sectioning, freeze etching analytical techniques, stereology, immune CM, histochemistry, autoradiography and, finally, the occupational hazards of electron microscopy, many of which have been overlooked for the last 2 decades.

The preface states that special emphasis has been placed on techniques where the beginner is apt to have difficulties, and this is borne out by an excellent chapter on sectioning, but the chapter on freeze etching gives no practical instruction whatsoever, being limited to 2 pages of text and 4 micrographs. Perhaps this will be covered more fully in a future volume. Other techniques are also rather briefly covered, and if this volume is intended as an introduction, it is insufficiently comprehensive, as techniques of plastic histology, which will become the main bridge between light and electron microscopy and also image analysis, are absent.

In general, however, the book makes a good attempt at providing an answer to the basic problem of EM laboratories being run by people without training in human pathology, and the general lack of ultrastructural experience among pathologists.

The books themselves are lavishly produced, being of large format and high-quality paper, with numerous photographs, and some colour plates. This is taken to excess in some areas, for example, 3 small photographs of ultratomes, which would easily have fitted on a single page and been a better comparison, have been separated on to 3 pages. In view of this, a question arises as to the cost effectiveness of this high-priced volume.

*Vol.* 2—*Cellular Pathobiology—Metabolic and Storage Diseases* (1979) 256 pp. £18.15.

The first part of this volume (by David, Berlin) deals with the structure and function of normal and diseased cells and organelles, thus serving as a basis for the later chapters on applied ultrastructural pathology. It is a lengthy portion (125 pp.) and provides an extremely useful catalogue of pathological alterations in ultrastructure of individual cellular organelles, with a detailed list of some 350 references.

Part two of the volume is devoted to metabolic and storage diseases, and begins with a useful introduction, technical and sampling section, before moving on to specific ultrastructural changes in metabolic diseases. This probably represents a unique grouping of ultrastructural pathologies such as Wolman's, Neuman-Pick's, Gauchier's, Fabry's, Tay-Sachs', Sandhoff's, Hurler's and Hunter's diseases, to Chediak-Higashi syndrome. These are laid out in a logical and clear manner, and profusely illustrated with high-quality micrographs. Volume 2 of this series has few of the faults of Volume 1, indicating that this series as a whole will be invaluable to those working with ultrastructure in the medical and paramedical fields.

T. D. ALLEN